# Fecal Arachidonic Acid: A Potential Biomarker for Inflammatory Bowel Disease Severity

**DOI:** 10.3390/ijms26094034

**Published:** 2025-04-24

**Authors:** Muriel Huss, Tanja Elger, Claudia Kunst, Johanna Loibl, Sabrina Krautbauer, Gerhard Liebisch, Arne Kandulski, Martina Müller, Hauke Christian Tews, Christa Buechler

**Affiliations:** 1 Department of Internal Medicine I, Gastroenterology, Hepatology, Endocrinology, Rheumatology, Immunology, and Infectious Diseases, University Hospital Regensburg, 93053 Regensburg, Germany; muriel.huss@klinik.uni-regensburg.de (M.H.); claudia.kunst@klinik.uni-regensburg.de (C.K.); johanna.loibl@klinik.uni-regensburg.de (J.L.); arne.kandulski@klinik.uni-regensburg.de (A.K.); martina.mueller-schilling@klinik.uni-regensburg.de (M.M.); hauke.tews@klinik.uni-regensburg.de (H.C.T.); 2 Institute of Clinical Chemistry and Laboratory Medicine, University Hospital Regensburg, 93053 Regensburg, Germany; sabrina.krautbauer@klinik.uni-regensburg.de (S.K.); gerhard.liebisch@klinik.uni-regensburg.de (G.L.)

**Keywords:** fatty acids, feces, calprotectin, stool consistency, inflammatory bowel disease, biomarker

## Abstract

Arachidonic acid levels are elevated in the colonic mucosa of patients with inflammatory bowel disease (IBD). Fecal metabolites are emerging as valuable diagnostic tools for IBD. This study aimed to investigate associations between 31 fecal fatty acids, including arachidonic acid, to identify potential correlations with disease severity. Among the 31 fatty acids analyzed in feces, dihomo-γ-linolenic acid, arachidonic acid, and adrenic acid were significantly increased in patients with IBD compared to controls. In contrast, levels of linoleic acid and γ-linolenic acid, the precursors of arachidonic acid, were similar between both groups. No significant differences in fatty acid levels were observed between patients with Crohn’s disease and ulcerative colitis. Arachidonic acid and adrenic acid levels positively correlated with fecal calprotectin, a clinically established marker of IBD severity, but showed no association with stool consistency or the Gastrointestinal Symptom Rating Scale. This suggests that these fatty acids are linked to disease severity rather than disease-related symptoms. Current IBD-specific medications had no significant impact on the fecal levels of any of the 31 fatty acids. In summary, this study demonstrates elevated fecal levels of dihomo-γ-linolenic acid, arachidonic acid, and adrenic acid in IBD patients. Normal levels of precursor fatty acids suggest that impaired downstream metabolism may contribute to the accumulation of these n-6 polyunsaturated fatty acids.

## 1. Introduction

The phrase “inflammatory bowel disease” (IBD) refers to a collection of long-lasting conditions, with the two most common being Crohn’s disease (CD) and ulcerative colitis (UC). In CD, chronic inflammation of the gastrointestinal mucosa can affect any part of the gastrointestinal tract, whereas in UC, it is confined to the colon and rectum. The cause of IBD is not well understood, but it is linked to environmental exposures that can provoke an abnormal immune response in those with a genetic predisposition [1,2,3,4,5].

Arachidonic acid is an n-6 polyunsaturated fatty acid that serves as a precursor for the synthesis of eicosanoids, leukotrienes, prostaglandins, and thromboxanes. These originate from lipoxygenase, cytochromes P450, phospholipase A2 or cyclooxygenase (COX) activity [6]. The enzyme COX-2 is induced in inflammatory processes, and the inhibition of COX-2 has been demonstrated to suppress the synthesis of prostaglandins and pain response [7]. However, studies have reported that COX-2 inhibition may be associated with relapse of IBD, and further data are required to clarify the potential adverse effects of these drugs [8].

Mucosal inflammation in patients with UC is typified by elevated levels of arachidonic acid [9]. Linoleic acid serves as the precursor for arachidonic acid synthesis, undergoing conversion to γ-linolenic acid, dihomo-γ-linolenic acid, and arachidonic acid [6]. The elevated mucosal levels of arachidonic acid and depletion of linoleic acid observed in inflamed compared to non-inflamed mucosa indicate that the synthesis of arachidonic acid is induced in active UC [9]. It is noteworthy that mucosal arachidonic acid levels are increased in correlation with higher endoscopic severity grades, whereas the decline of linoleic acid was not statistically significant [9]. The levels of γ-linolenic acid and dihomo-γ-linolenic acid were not determined in that study [9]. However, it was reasonable to suggest that arachidonic acid synthesis from linoleic acid is increased in mucosal inflammation [9].

Lipid profiling of epithelial cells from the inflamed mucosa of UC patients revealed a decline in phospholipids containing mono- and di-unsaturated fatty acids and higher levels of arachidonic acid-containing species in comparison to non-inflamed tissue [10]. In the inflamed epithelium, enzymes that catalyze the synthesis of arachidonic acid-derived thromboxanes and prostaglandins were induced [11,12].

IBD is associated with an imbalance of the intestinal microbiota, and dysbiosis contributes to altered levels of different lipid species in the feces of these patients. Accordingly, the composition of short-chain fatty acids and bile acids in the feces of IBD patients differs from that of healthy controls [13,14,15,16]. However, to the best of our knowledge, there is only one study, which measured six different fatty acids including oleic acid, stearic acid, palmitic acid and arachidonic acid, in stool of eight patients with colonic CD and six patients with ileal CD and 20 healthy controls, and described higher levels of the fatty acids in patients with ileal CD [17]. However, a comprehensive analysis of fecal fatty acid levels has yet to be performed in IBD.

Although most studies have focused on the pro-inflammatory effects of n-6 polyunsaturated fatty acids, such as dihomo-γ-linolenic acid and arachidonic acid, these fatty acids and their metabolites also play a role in the maintenance of the epithelial barrier provided by intestinal epithelial cells. Furthermore, they have been demonstrated to regulate proliferation, facilitate intestinal regeneration, and exert anti-inflammatory effects [18,19,20].

Several studies have examined the impact of dietary lipids on intestinal inflammation [21]. In Western populations, a higher intake of n-6 polyunsaturated fatty acids has been linked to an increased risk of UC, while in Eastern populations, a higher intake of total fat, monounsaturated fatty acids, and both n-3 and n-6 polyunsaturated fatty acids has been associated with an increased risk of CD [22].

Fecal proteins have emerged as promising biomarkers for IBD, with fecal calprotectin already established as a non-invasive marker of mucosal inflammation in clinical practice [23,24]. Fecal calprotectin measurement has limitations, and its positive predictive value is less than 10% [25]. Fecal calprotectin levels are induced in various diseases and are also elevated in colorectal cancer [26,27]. Most fecal fatty acids, including arachidonic acid, in patients with colorectal cancer were not different from the healthy controls [28]. Plasma long-chain fatty acids, such as arachidonic acid, were either normal or reduced in IBD as compared to healthy controls [29,30,31], whereas serum calprotectin levels were increased [32]. This indicates that calprotectin and long-chain fatty acid levels are differentially altered in IBD, and a combination may diagnose IBD and monitor disease activity with greater accuracy.

Combinations of appropriate biomarkers usually improve the diagnostic sensitivity and specificity and may allow early diagnosis of IBD, which is essential for effective therapy [33,34]. It is currently unclear whether fecal lipids are altered in IBD and whether this is associated with disease severity. Fecal arachidonic acid is thought to be derived from the diet [35], but also from damaged intestinal cells, suggesting a close association with IBD severity.

The potential of fecal fatty acid analysis as a diagnostic tool remains unexplored. This study investigates whether fecal fatty acid profiling can serve as a reliable biomarker for IBD diagnosis and disease monitoring.

## 2. Results

### 2.1. Fecal Fatty Acid Levels in Patients with IBD and Controls

The study cohort consisted of 62 patients diagnosed with inflammatory bowel disease (IBD), comprising 38 patients with Crohn’s disease (CD) and 24 patients with ulcerative colitis (UC), along with 17 healthy controls (Table 1). The controls and patients with IBD did not show significant differences in age and sex distribution (Table 1). All controls were in good condition and within normal weight parameters, and body mass index (BMI) and laboratory measures were not recorded.

The total fatty acid content in the dry weight of stool was comparable between IBD patients and healthy controls. Similarly, saturated, monounsaturated, di-unsaturated, and polyunsaturated fatty acid levels showed no significant differences between the two groups, with the fatty acyl chain composition also remaining consistent (*p* > 0.05 for all).

**Figure 1:**
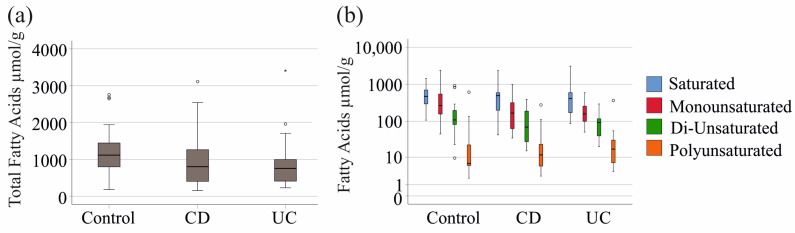
Fecal fatty acid levels (µmol/g dry weight) of controls, patients with Crohn’s Disease (CD), and patients with ulcerative colitis (UC). (**a**) Total levels of fecal fatty acids were similar between the groups; (**b**) Concentrations of saturated, monounsaturated, di-unsaturated, and polyunsaturated fatty acids in stool of controls and patients were similar between the groups. Boxplots highlight outliers with circles and asterisks.

No significant differences were observed between CD and UC patients in terms of total fatty acid levels or saturated, monounsaturated, di-unsaturated, and polyunsaturated fatty acid composition (Figure 1a,b).

The most abundant fatty acids in stool were oleic acid (median 254.40 µmol/g (controls), 146.40 µmol/g (IBD)) and palmitic acid (median 200.46 µmol/g (controls), 237.45 µmol/g (IBD)), followed by stearic acid (median 167.70 µmol/g (controls), 150.15 µmol/g (IBD)) and linoleic acid (median 108.29 µmol/g (controls), 81.10 µmol/g (IBD)). Levels of myristic acid were approximately 11 µmol/g in controls and 15 µmol/g in IBD, while all other fatty acids exhibited median levels below 10 µmol/g (Table 2).

The 31 fatty acids in the stool of male and female controls and patients with IBD were similar (*p* > 0.05 for all). Furthermore, no correlation was observed between fecal fatty acid levels and age or BMI in patients with IBD (*p* > 0.05 for all).

A comparison of patients with IBD and controls revealed that fecal dihomo-γ-linolenic acid, arachidonic acid, and adrenic acid, whose chemical structure is shown in Figure S1, were present at higher levels in IBD patients than in controls (Table 2). A comparison of patients with CD and UC showed no significant differences in the levels of the analyzed fatty acids (*p* > 0.05 for all).

**Figure 2:**
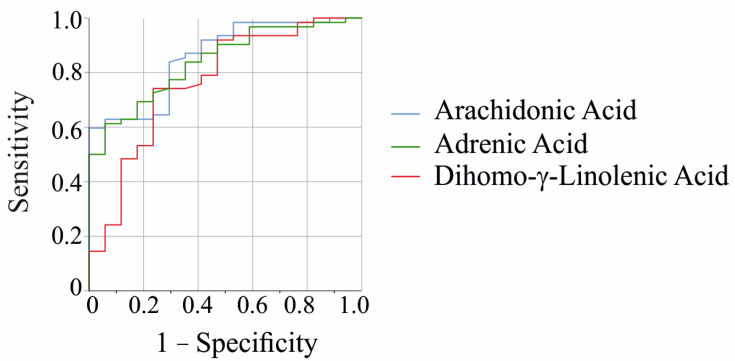
Receiver operating characteristic curve for the discrimination of patients and controls by fecal arachidonic acid, adrenic acid, and dihomo-γ-linolenic acid. The other fatty acids measured in feces were similar between patients and controls.

A receiver operating characteristic (ROC) curve analysis revealed that dihomo-γ-linolenic acid (area under the ROC curve (AUROC) 0.769 ± 0.069, *p* = 0.022), arachidonic acid (AUROC 0.859 ± 0.046, *p* < 0.001), and adrenic acid (AUROC 0.841 ± 0.047, *p* < 0.001) were found to be effective in discriminating patients from controls (Figure 2).

### 2.2. Fecal Levels of Arachidonic Acid and Adrenic Acid Increase with Fecal Calprotectin, but Show No Correlation with CRP or Markers of Renal Function

Spearman correlation analyses were conducted to assess the relationship between fecal fatty acids and fecal calprotectin. Most fecal fatty acids did not correlate significantly with fecal calprotectin, a clinical marker for intestinal inflammation and IBD activity monitoring [36,37]. However, arachidonic acid (r = 0.507, *p* < 0.001) and adrenic acid (r = 0.480, *p* < 0.01) exhibited a positive correlation with fecal calprotectin.

**Figure 3:**
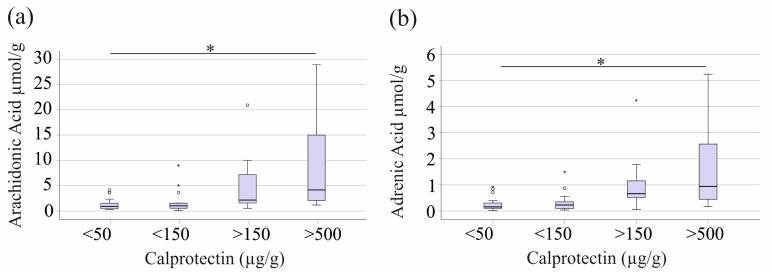
Connection between fecal fatty acid and fecal calprotectin levels. Concentrations (µmol/g dry weight) of (**a**) arachidonic acid and (**b**) adrenic acid in stool of IBD patients classified by fecal calprotectin levels. * *p* < 0.05. Boxplots highlight outliers with circles and asterisks. Arachidonic acid and adrenic acid, but none of the other fecal fatty acids measured, increased with higher fecal calprotectin levels.

Of the patients with IBD, 25 patients had fecal calprotectin levels below 50 µg/g, 20 had levels from 50 to <150 µg/g, eight had levels from 150 to 500 µg/g, and eight had levels above 500 µg/g. Data for one patient were unavailable. Arachidonic acid (*p* = 0.017) and adrenic acid (*p* = 0.012) showed higher levels in patients with calprotectin > 150 µg/g and significantly elevated concentrations in those with >500 µg/g compared to patients with low (<50 µg/g) fecal calprotectin (Figure 3).

No correlation was found between serum CRP and fecal fatty acids. This indicates that fecal fatty acids are not strongly related to systemic inflammation, and associations with inflammatory cytokines are most likely not significant.

Fecal fatty acid levels showed no association with creatinine or glomerular filtration rate (GFR) (*p* > 0.05 for all).

In this study, a fecal calprotectin concentration >120 µg/g was considered indicative of IBD diagnosis. It was shown through ROC curve analysis that fecal arachidonic acid at a threshold of 1.63 µmol/g could distinguish patients with calprotectin levels above and below this cut-off, achieving a sensitivity of 72% and a specificity of 77% (AUROC = 0.783, *p* = 0.016). Similarly, adrenic acid at a cut-off of 0.46 µmol/g distinguished these groups with 67% sensitivity and 84% specificity (AUROC = 0.785, *p* = 0.015).

Notably, arachidonic acid levels were significantly higher in IBD patients with calprotectin < 50 µg/g compared to controls (*p* = 0.028). However, adrenic acid (*p* = 0.143) and dihomo-γ-linolenic acid (*p* = 0.539) levels did not show significant differences between these groups.

### 2.3. Correlations of Fecal Fatty Acids with Serum Cholesterol and Triglyceride Levels

Inflammation in IBD is related to low serum cholesterol levels, whereas the data for serum triglycerides are inconsistent [38,39,40,41]. Serum cholesterol (*p* = 0.800) and triglyceride levels (*p* = 0.243) of the 16 controls and 58 patients for whom these measurements were available did not differ between patients with IBD and controls. Serum cholesterol levels, but not triglyceride levels, were negatively correlated with fecal calprotectin and CRP in patients with IBD (Table S1). None of the fecal fatty acids correlated with serum triglyceride levels (Table S1). Fecal arachidonic acid was negatively correlated with total serum cholesterol levels (Table S1). This shows that fecal arachidonic acid in IBD is associated with inflammation-induced dyslipidemia rather than the routinely measured marker of inflammation, CRP.

### 2.4. Associations of Fecal Fatty Acids with the Bristol Stool Chart and Gastrointestinal Symptom Rating Scale

The relationship between fecal fatty acid levels, stool consistency, and Gastrointestinal Symptom Rating Scale (GSRS) scores was also assessed. Patients recorded the consistency of their stool sample, which was classified according to the Bristol Stool Chart:

1. Constipation (Types 1 and 2): 5 patients.
2. Normal (Types 3 and 4): 15 patients.
3. Diarrhea (Types 5 and 6): 33 patients.
4. Watery Stool (Type 7): 9 patients.

**Figure 4:**
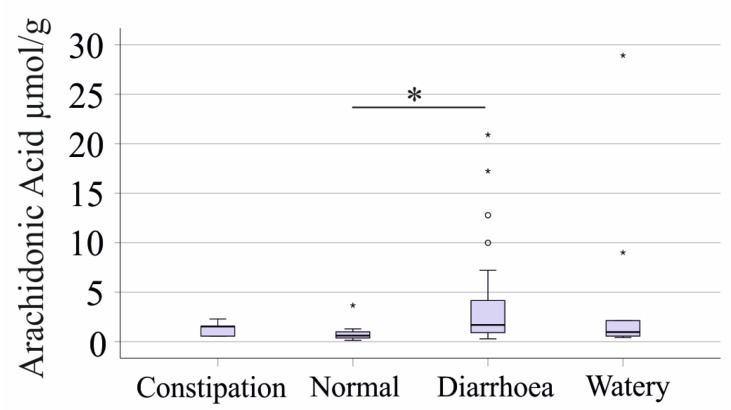
Levels of fecal arachidonic acid (µmol/g dry weight) according to stool consistency. Patients with diarrhea have higher fecal levels of arachidonic acid compared to patients with normal stool consistency * *p* < 0.05. All of the other fatty acids did not differ between the groups. Boxplots highlight outliers with circles and asterisks.

Patients with diarrhea exhibited significantly higher fecal arachidonic acid levels compared to those with normal stool (*p* = 0.024, Figure 4). Similarly, fecal calprotectin levels tended to be higher in diarrhea patients than in those with normal stool (*p* = 0.057). CRP of patients with diarrhea was significantly higher (*p* = 0.014) than that of patients with normal stool.

However, the differences between normal and watery stool groups were not statistically significant, likely due to the small sample size in the watery stool group.

Figure S2 shows the fatty acids (fatty acids were classified as saturated, monounsaturated, di-unsaturated, and polyunsaturated and were also classified according to the number of carbon atoms) of patients with different stool consistencies. Normal levels of almost all fatty acids in patients with diarrhea show that normalization of fatty acid levels by stool dry weight is appropriate to account for the water content of stool.

The GSRS, which evaluates gastrointestinal symptoms, showed no significant association between fecal fatty acid levels and symptom scores (*p* > 0.05 for all).

### 2.5. Fecal Fatty Acids in Relation to Time Since First Diagnosis and Disease Localization

The time since IBD diagnosis, recorded for 60 patients, ranged from 1 to 42 years, with a median of 10 years. However, no correlation was found between disease duration and any fecal fatty acid species.

In the CD group, six patients had isolated ileocecal involvement, while 30 patients had ileocecal involvement along with other affected gastrointestinal regions. Additionally, two patients exhibited colonic inflammation. Fecal fatty acid levels did not significantly differ between these subgroups (*p* > 0.05 for all).

Among UC patients, 13 had pancolitis, five had left-sided colitis, three had proctosigmoiditis, and two had isolated proctitis. Data of one patients were not recorded. Similarly, no significant differences in fecal fatty acid levels were observed between these subgroups (*p* > 0.05 for all).

### 2.6. Effects of Medication on Fecal Fatty Acid Composition

The potential impact of current IBD therapy on fecal fatty acid levels was also assessed. Among the patients: 22 received anti-TNF therapy, 17 were treated with corticosteroids, and 18 received anti-IL-12/23 antibodies.

None of these treatments were associated with significant changes in fecal fatty acid levels (*p* > 0.05 for all), suggesting that fecal fatty acid composition remains unaffected by different therapies. Figure S3 shows the fatty acid composition (fatty acids were classified as saturated, monounsaturated, di-unsaturated, and polyunsaturated and were also classified according to the number of carbon atoms) of patients treated with the above therapies compared to patients with the other treatments.

## 3. Discussion

Our study demonstrates that fecal dihomo-γ-linolenic acid, arachidonic acid, and adrenic acid levels are elevated in individuals with IBD compared to healthy controls. Notably, arachidonic acid and adrenic acid showed a positive association with intestinal inflammation, as indicated by fecal calprotectin levels.

To our knowledge, a comprehensive analysis of fecal fatty acid levels in IBD patients has not been previously documented. Among all fecal fatty acids, oleic acid and palmitic acid were the most abundant. Linoleic acid, an essential dietary fatty acid, was detected in the control group at approximately half the concentration of palmitic acid.

Impaired bile acid metabolism and terminal ileum inflammation in CD can lead to fatty acid malabsorption [42,43]. However, 28 of the 31 fecal fatty acids measured in this study showed no significant differences between IBD patients and controls. Additionally, fecal fatty acid profiles were similar between patients with CD and patients with UC, suggesting that IBD does not substantially alter fecal fatty acid composition. Despite this overall similarity, fecal dihomo-γ-linolenic acid, arachidonic acid, and adrenic acid were elevated in IBD patients. These fatty acids are derived from linoleic acid and γ-linolenic acid [6] both of which remained within normal ranges in IBD patients.

In serum, patients with CD showed increased proportions of dihomo-γ-linolenic acid, eicosapentaenoic acid, docosapentaenoic acid, and oleic acid, whereas arachidonic acid levels were lower compared to controls [44]. Neutrophils of patients with IBD release lower levels of arachidonic acid and higher levels of its 5-lipoxygenase products [45], which may contribute to the reduced systemic levels of arachidonic acid. These data suggest an increased metabolism of arachidonic acid, but cannot exclude that increased fecal excretion may also play a role in the low serum arachidonic acid levels in patients with CD.

In the inflamed mucosa of UC patients, arachidonic acid levels were increased, whereas linoleic acid levels were reduced, suggesting induced arachidonic acid synthesis [9]. A subsequent study found that colonic mucosal phospholipids in both UC and CD contained higher arachidonic acid levels than controls, while linoleic acid, oleic acid, palmitic acid, and stearic acid levels remained unchanged [46]. These findings are consistent with our fecal analysis, where most fatty acids showed no significant differences between IBD patients and controls. However, while fecal arachidonic acid levels correlated with disease severity, mucosal arachidonic acid levels were not directly linked to inflammation [46].

The normal levels of fatty acid precursors for dihomo-γ-linolenic acid synthesis suggest that its processing, rather than production, is altered in IBD. Dihomo-γ-linolenic acid, a precursor of arachidonic acid, appears to be efficiently converted, as arachidonic acid levels are increased in IBD. Additionally, adrenic acid, which is derived from arachidonic acid, is also elevated. The conversion of arachidonic acid to eicosapentaenoic acid and dihomo-γ-linolenic acid to eicosatetraenoic acid, which are both catalyzed by delta-17-desaturase [47] appears to be normal as the levels of these two omega-3 fatty acids were not altered.

Several highly bioactive lipid mediators originate from dihomo-γ-linolenic acid, arachidonic acid, and adrenic acid. These include prostaglandins, thromboxanes, leukotrienes, hydroxyeicosatetraenoic acids (HETEs), epoxy fatty acids, and lipoxins [19,20,48]. Although these metabolites were not measured in the present study, it is plausible that their synthesis is dysregulated in IBD. However, further analyses of these highly active metabolites are needed to prove this suggestion. Previous studies have reported positive correlations between colonic mucosal inflammation in UC and levels of prostaglandin E2 and D2, thromboxane A2, and 5-, 11-, 12-, and 15-HETE [49]. Furthermore, higher levels of 12S-HETE in inactive CD compared to active CD and increased concentrations of LTX A4, 5S, and 6R in inactive UC compared to active UC have been observed [50]. A comprehensive analysis of lipid mediators is needed to confirm the hypothesis that impaired metabolite production contributes to fecal arachidonic acid accumulation in active IBD.

At a cut-off concentration of 1.63 µmol/g, fecal arachidonic acid distinguished patients with calprotectin levels < 120 µg/g and >120 µg/g, demonstrating 72% sensitivity and 77% specificity. At a threshold of 0.46 µmol/g, adrenic acid demonstrated a sensitivity of 67% and specificity of 84%. Fecal calprotectin at a threshold of 50 µg/g showed a sensitivity of 93% for IBD diagnosis, but its specificity was lower at 62%. [25]. However, our cohorts were small, and further studies in larger cohorts are needed to validate the specificity and sensitivity of these fecal metabolites for the diagnosis of IBD.

Fecal calprotectin is a marker of inflammation and is also increased in colorectal cancer [26,27]. Arachidonic acid levels in patients with colorectal cancer were normal, which is an advantage when using this as a biomarker for IBD diagnosis [28]. Serum calprotectin levels were elevated in patients with IBD [32], whereas arachidonic acid in the plasma of patients with CD was similar to that in healthy controls [29] and was also found to be lower compared to controls [44]. Fecal calprotectin is positively related to systemic inflammation, which is not the case for fecal arachidonic acid. Therefore, fecal arachidonic acid seems to be a relatively specific marker of intestinal inflammation.

Fecal arachidonic acid, fecal calprotectin, and serum CRP were all negatively correlated with serum cholesterol levels. Inflammation in IBD is associated with a decrease in serum cholesterol levels [38,39,40,41] consistent with the negative associations of serum cholesterol with CRP and calprotectin. Fecal arachidonic acid did not correlate with CRP but was negatively associated with serum cholesterol levels. Although the underlying pathways are unknown, this shows that fecal arachidonic acid levels are related to inflammation-induced changes in cholesterol metabolism rather than to clinical markers of inflammation.

Combinations of appropriate biomarkers usually improve the diagnostic sensitivity and specificity and may allow early diagnosis of IBD, which is essential for effective therapy [33,34]. Fecal arachidonic acid is thought to be mainly derived from damaged epithelial cells, suggesting a close association with IBD. The diagnosis of IBD and the monitoring of disease activity may be improved by measuring fecal arachidonic and/or adrenic acid in addition to fecal calprotectin. As fecal calprotectin serves as a general marker of intestinal inflammation and not specifically for IBD [24,37], the combination with other markers is advantageous. It should also be noted that arachidonic acid in feces was higher in patients with <50 µg/g calprotectin compared to healthy controls, suggesting that fecal arachidonic acid is already elevated in mild cases. Studies in larger cohorts of patients with different gastrointestinal diseases are needed to confirm these suggestions. This multi-center study should enroll IBD patients and healthy controls of both sexes with a wide range of BMI, age, and disease activity. Patients with other gastrointestinal diseases, such as irritable bowel syndrome, should also be included. Most of our patients with CD had ileocecal disease, and future analysis is needed to clarify the associations of fecal fatty acids with disease localization. Higher fecal levels of oleic acid, stearic acid, palmitic acid, and arachidonic acid have been described in patients with ileal CD. However, this cohort was small [17], and confirmation is needed in much larger studies.

Stool fatty acids were normalized to the dry weight of the samples, and this approach seems appropriate for comparing fecal biomarkers between IBD patients with different disease severity and stool consistency, as most fecal fatty acids were similar between these groups. The majority of fecal fatty acids showed no association with stool consistency, excluding steatorrhea as a contributing factor to diarrhea in our patients [51]. Patients with diarrhea had slightly elevated fecal arachidonic acid levels, calprotectin, and CRP compared to those with normal stools, reflecting higher disease activity in this group, which has been described as a contributing factor to diarrhea [51].

Age, sex, and current medication did not affect the diagnostic performance of fecal arachidonic and adrenic acid, reinforcing their potential as reliable biomarkers. However, the association of different therapies on fecal fatty acid levels has to be studied in a larger cohort of treatment-naïve patients to finally exclude that specific medications have an effect.

Diet modulates fatty acid stool composition, but studies analyzing fecal fatty acid composition in healthy people in relation to specific diets are rare. One study involving healthy young adults showed that a high-fat diet (40% fat) for 6 months increased fecal levels of palmitic, stearic, and arachidonic acid compared to a low-fat diet (20% fat) cohort [52]. Furthermore, there are few studies that have compared the fecal fatty acid composition of lean and obese individuals. Lean controls had higher fecal linolenic and linoleic acids and lower fecal arachidonic, adrenic, and dihomo-γ-linolenic acid levels compared to obese individuals [53]. This study did not provide evidence that dietary composition differed significantly between the obese and lean cohorts [53]. Further studies are needed to evaluate the associations of fecal fatty acid levels with diet and BMI. It should be noted that obesity is also associated with higher fecal calprotectin levels [54] but this does not seem to limit its diagnostic value [55].

This study has several limitations. Fecal lipid metabolites of dihomo-γ-linolenic acid, arachidonic acid, and adrenic acid were not analyzed. Serum fatty acid composition was also not determined. Additionally, as a single-center study conducted in Bavaria, the findings may not be fully generalizable to other regions of Germany or other countries. Stool samples were collected during the day without fasting, and dietary habits were not documented, which could influence fecal fatty acid levels. Clinical scores for IBD were not included, and clinical data for the patients are incomplete. Laboratory values and BMI of the controls, which were few, were not included, but all controls were of normal body weight and good health.

## 4. Materials and Methods

### 4.1. Patients

Patients aged 18 or older with IBD were enrolled in the study from 6 December 2021 to 31 January 2023, at the outpatient and inpatient clinics of the Department of Internal Medicine I, at the University Hospital Regensburg. Participants were randomly selected from our tertiary care facility, and all patients interested in participating in the study were included. The diagnosis of IBD was validated by a combination of histological analysis, endoscopic procedures, and clinical assessments [56]. The study excluded patients with a diagnosis of coagulopathy, people with primary sclerosing cholangitis, and pregnant women.

Endoscopic scores were not consistently assessed at the times of blood collection, and were not included in this analysis.

A G*Power 3.1.6. analysis [57] using the mean values and standard deviation of arachidonic acid of patients with IBD and controls revealed that 16 controls and 62 patients are sufficient (alpha-error = 0.05, power = 80%) for this study.

### 4.2. Stool Collection, Fecal Fatty Acid, and Calprotectin Analysis

Stool samples from patients and controls (hospital staff, students, and partners of the patients) were obtained in 70% isopropanol. These samples were kept at −80 °C until they were processed. A GentleMACS™ Dissociator (Miltenyi Biotec GmbH, Bergisch Gladbach, Germany) was used to carry out the homogenization.

For the determination of dry weight, vacuum centrifugation was used to dry 1.0 mL of homogenized stool. For further analysis, the homogenates were diluted to a final concentration of 2.0 mg/mL of dry weight.

Quantification of fatty acids: Gas chromatography-mass spectrometry (GC-MS) was employed after the samples were derivatized into fatty acid methyl esters (FAMEs), using a modified protocol by Ecker et al. [58] with following GC-MS conditions: Initial column temperature: 50 °C (0.75 min); Temperature ramp: 40 °C/min to 110, with 6 °C/min to 210, with 15 °C/min to 250 °C, held for 2 min. Quantification was performed using calibration lines consisting of four levels generated by dilutions of an authentic FAME standard (FAME Mix 37, Supelco—Merck, Taufkirchen, Germany), and a constant level of FAME 21:0iso was used as an internal standard. Selected ion monitoring was applied, and FAME 20:4-c5,c8,c11,c14 (n-6) was quantified with the fragment *m*/*z* 79 relative to the internal standard FAME 21:0iso.

The limit of detection was established using diluted standard samples, specifically looking for a signal-to-noise ratio of 3. The limit of detection ranged from 1.9 to 16 fmol on column, with an increase noted alongside longer fatty acid chain lengths and higher degrees of unsaturation. The fatty-acid FAME peaks in the fecal samples far exceeded a signal-to-noise ratio of 3. Data acquisition and processing used the GC–MS Solution Software from Shimadzu (Version 4.45; Kyoto, Japan) [58].

Quantification of serum cholesterol and serum triglyceride levels was carried out as described [59,60]. Fecal calprotectin was measured using Quanta Flash Calprotectin (Inova Diagnostics, San Diego, CA, USA).

### 4.3. Statistical Analysis

Details of the statistical analysis and presentation of data are summarized in Table 3.

## 5. Conclusions

Our study found that most fecal saturated, monounsaturated, and polyunsaturated fatty acid levels in IBD patients were comparable to those in healthy controls. Of clinical relevance, dihomo-γ-linolenic acid, arachidonic acid, and adrenic acid showed elevated levels, with arachidonic acid and adrenic acid correlating with disease activity. This suggests a potential role in mucosal inflammation and highlights their value as additional biomarkers for monitoring disease severity in clinical practice.

## Figures and Tables

**Table 1:** Features of the patients with IBD and the healthy control group. The sex distribution and age of these two cohorts were comparable.

Characteristics	IBD	Controls
Number (female/male)	62 (28/34)	17 (10/7)
Age (years)	42 (19–78)	48 (23–78)
Body Mass Index (kg/m^2^)	24 (16–44)	not determined
C-reactive protein (mg/L)	3 (0–144)	not determined
Creatinine (mg/dL)	0.85 (0.51–1.25)	not determined
Glomerular filtration rate (mL/min)	99 (61–136)	not determined
Fecal calprotectin (µg/g)	62 (17–1616)	not determined

**Table 2:** Median, minimum, and maximum fatty acid levels (µmol/g) in feces of controls and patients with IBD. Significant different fatty acid levels are given in bold. Not significant, ns.

Fatty Acid (µmol/g Dry Weight)	Controls			IBD			
	Median	Minimum	Maximum	Median	Minimum	Maximum	*p*-Value
FA8:0 (Caprylic Acid)	0.27	0.05	3.09	0.33	0.06	3.02	ns
FA10:0 (Capric Acid)	0.47	0.20	3.29	0.52	0.09	4.70	ns
FA11:0 (Undecyclic Acid)	0.10	0.04	0.39	0.10	0.00	0.83	ns
FA12:0 (Lauric Acid)	2.34	0.88	16.58	3.01	0.45	801.77	ns
FA14:0 (Myristic Acid)	10.63	4.69	31.39	14.84	1.60	348.33	ns
FA14:1 c9	0.21	0.11	1.34	0.31	0.00	5.88	ns
FA15:0 (Pentadecyclic Acid)	6.89	2.33	18.69	6.37	0.38	21.22	ns
FA16:0 (Palmitic Acid)	200.46	54.80	474.02	237.45	24.56	950.08	ns
FA16:1:c9 (Palmitoleic Acid)	3.46	1.06	10.08	3.68	0.98	23.73	ns
FA17:0 (Margaric Acid)	3.95	1.49	17.61	3.94	0.38	23.81	ns
FA18:0 (Stearic Acid)	167.70	35.14	1008.66	150.15	12.15	1600.20	ns
FA18:1:c9:n-9 (Oleic Acid)	254.40	24.93	2352.78	146.40	23.63	946.50	ns
FA18:1 c11:n-7 (Cis-Vaccenic Acid)	7.34	1.58	17.71	5.55	0.92	25.75	ns
FA18:2:t9t12:n-6 (Linolelaidic Acid)	0.76	0.05	5.02	0.27	0.00	4.57	ns
FA18:2:c9c12:n-6 (Linoleic Acid)	108.29	8.93	916.03	81.10	14.71	391.85	ns
FA18:3:c6c9c12:n-6 (γ-Linolenic Acid)	0.03	0.01	0.63	0.05	0.00	0.70	ns
FA18:3 c9c12c15 n-3 (α-Linolenic acid)	3.21	0.63	611.23	4.06	0.32	363.19	ns
FA20:0 (Arachidic Acid)	5.36	0.92	12.76	3.82	0.51	50.87	ns
FA20:1 c11n-9 (Gondoic Acid)	1.82	0.70	8.69	2.16	0.59	26.45	ns
FA20:2:11c14c (Eicosadienoic Acid)	0.23	0.10	0.51	0.42	0.07	2.78	ns
FA21:0 (Heneicosylic Acid)	0.21	0.08	0.34	0.23	0.06	0.99	ns
**FA20:3 c8c11c14 n-6** **(Dihomo-γ-Linolenic Acid)**	**0.15**	**0.00**	**3.27**	**0.81**	**0.06**	**10.50**	**0.022**
**FA20:4 c5c8c11c14 n-6 (Arachidonic Acid)**	**0.33**	**0.12**	**0.93**	**1.22**	**0.13**	**28.91**	**<0.001**
FA20:3 c11c14c17 n-3 (Eicosatrienoic Acid)	0.05	0.01	0.44	0.08	0.01	1.19	ns
FA22:0 (Behenic Acid)	3.88	0.60	18.01	2.52	0.85	28.08	ns
FA20:4 c8c11c14c17 n-3 (Eicosatetraenoic Acid)	0.05	0.00	0.49	0.04	0.01	3.15	ns
FA22:1 c13 n-9 (Erucic Acid)	0.54	0.21	3.10	1.18	0.26	7.70	ns
FA20:5 c5c8c11c14c17 n-3 (Eicosapentaenoic Acid)	0.12	0.04	0.67	0.12	0.03	10.01	ns
FA22:2 c13c16	0.12	0.05	0.32	0.14	0.03	0.33	ns
FA23:0 (Tricosylic Acid)	0.59	0.17	1.20	0.57	0.15	2.55	ns
**FA22:4 7c10c13c16c** **(Adrenic Acid)**	**0.07**	**0.02**	**0.30**	**0.28**	**0.02**	**5.24**	**<0.001**

**Table 3:** Data presentation and statistical test used in the current study.

**Statistical tests applied**	Mann–Whitney U-test (two-group comparisons), Kruskal–Wallis test (multiple group comparisons), Spearman correlation (associations between variables), ROC analysis (diagnostic performance assessment), Youden’s statistic (optimal cut-off values)
**Data are presented as**	Boxplots (median, minimum, maximum).
**Outlier identification**	Boxplots highlight outliers with circles and asterisks
**Normality testing**	Kolmogorov–Smirnov and Shapiro–Wilk tests (*p* < 0.05 for all) indicated a non-normal distribution
**Correction for multiple comparisons**	*p*-values were adjusted by multiplying by 31 (total number of fatty acids analyzed).
**Significance threshold**	*p* < 0.05
**Table presentation**	Data are reported as median, minimum, and maximum values
**Software used**	IBM SPSS Statistics 26.0 (IBM Corp., Armonk, NY, USA, released 2019)

## Data Availability

Original research data can be obtained on request.

## Supplementary Materials

The following supporting information can be downloaded at: https://www.mdpi.com/article/10.3390/ijms26094034/s1.
