# Supplementary material for: Fecal Arachidonic Acid: A Potential Biomarker for Inflammatory Bowel Disease Severity
Source: Int J Mol Sci. 2025 Apr 24;26(9):4034. doi: 10.3390/ijms26094034 (PMC12071911; doi:10.3390/ijms26094034)
Supplement: Supplementary file 1 [file ijms-26-04034-s001.zip › ijms-3485965-supplementary.pdf]

Fecal Arachidonic Acid: A Potential Biomarker for Inflammatory Bowel Disease Severity

Muriel Huss [1], Tanja Elger [1], Claudia Kunst [1], Johanna Loibl [1], Sabrina Krautbauer [2], Gerhard Liebisch [2], Arne Kandulski [1], Martina Müller [1], Hauke Christian Tews [1] and Christa Buechler [1,*]

Table S1. Spearman correlation coefficients for the correlation of total serum cholesterol and triglyceride levels with fecal fatty acids. Significant correlations are in bold, and the p-values are given in the table.

	Serum Cholesterol	Serum Triglycerides
FA8:0 (Caprylic Acid)	-0.152	-0.099
FA10:0 (Capric Acid)	0.068	0.198
FA11:0 (Undecyclic Acid)	0.074	0.052
FA12:0 (Lauric Acid)	0.145	0.278
FA14:0 (Myristic Acid)	0.145	0.308
FA14:1 c9	0.024	0.197
FA15:0 (Pentadecyclic Acid)	0.130	0.209
FA16:0 (Palmitic Acid)	0.053	0.160
FA16:1:c9 (Palmitoleic Acid)	0.102	0.144
FA17:0 (Margaric Acid)	0.159	0.197
FA18:0 (Stearic Acid)	0.323	0.131
FA18:1:c9:n-9 (Oleic Acid)	0.145	0.068
FA18:1 c11:n-7 (Cis-Vaccenic Acid)	0.137	0.076
FA18:2:t9t12:n-6 (Linolelaidic Acid)	0.160	0.067
FA18:2:c9c12:n-6 (Linoleic Acid)	0.014	-0.018
FA18:3:c6c9c12:n-6 (γ-Linolenic Acid)	-0.181	0.141
FA18:3 c9c12c15 n-3 (α-Linolenic acid)	0.093	0.084
FA20:0 (Arachidic Acid)	0.302	0.165
FA20:1 c11n-9 (Gondoic Acid)	0.019	0.203
FA20:2:11c14c (Eicosadienoic Acid)	-0.338	-0.128
FA21:0 (Heneicosylic Acid)	0.165	0.096
FA20:3 c8c11c14 n-6 (Dihomo-γ-Linolenic Acid)	0.371	0.077
FA20:4 c5c8c11c14 n-6 (Arachidonic Acid)	**-0.518**<br>**p = 0.0042**	-0.062
FA20:3 c11c14c17 n-3 (Eicosatrienoic Acid)	-0.044	0.186
FA22:0 (Behenic Acid)	0.130	0.137
FA20:4 c8c11c14c17 n-3 (Eicosatetraenoic Acid)	-0.265	0.113
FA22:1 c13 n-9 (Erucic Acid)	-0.283	-0.170
FA20:5 c5c8c11c14c17 n-3 (Eicosapentaenoic Acid)	-0.347	-0.200
FA22:2 c13c16	-0.023	-0.039
FA23:0 (Tricosylic Acid)	0.181	0.158
FA22:4 7c10c13c16c (Adrenic Acid)	-0.420	-0.062
Fecal Calprotectin	**-0.468**<br>**p = 0.007**	-0.259
CRP	**-0.421**<br>**p = 0.047**	-0.116

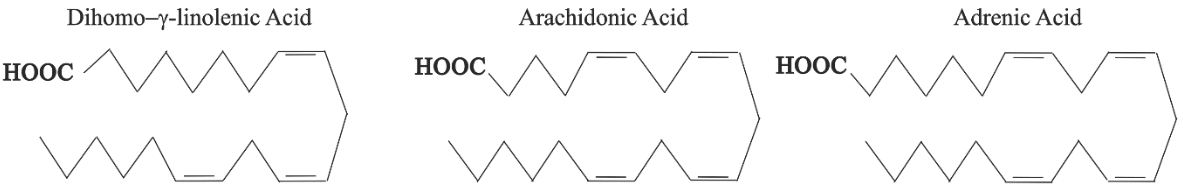

Figure S1. Chemical structure of dihomo-γ-linolenic acid, arachidonic acid and adrenic acid.

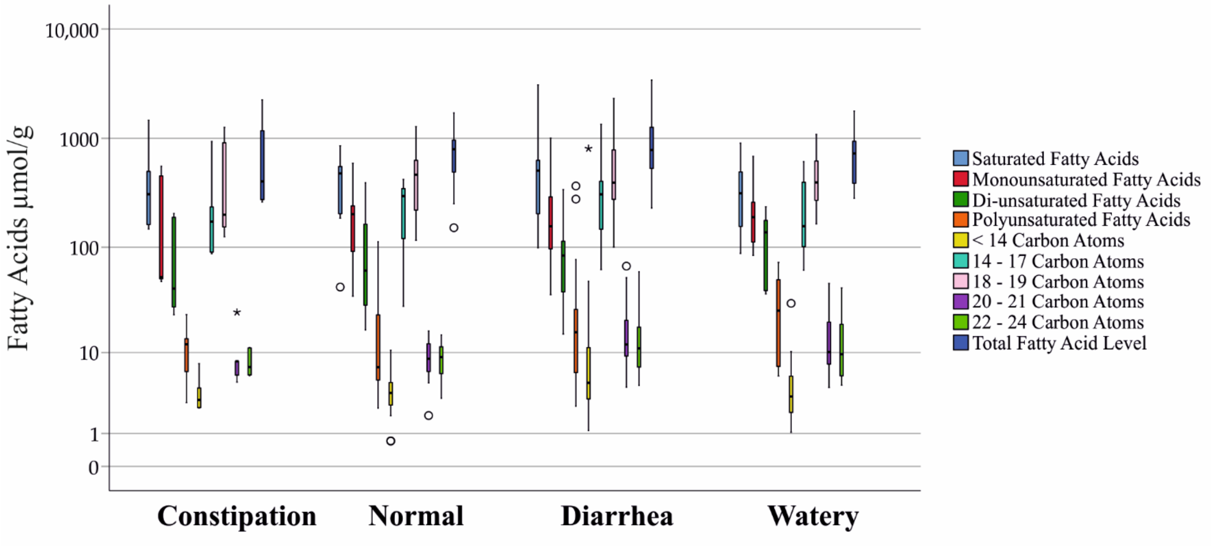

Figure S2. Saturated, monounsaturated, di-unsaturated and polyunsaturated fatty acid levels and fatty acid levels classified according to the number of carbon atoms of patients with different stool consistencies. There were no differences between the groups. Boxplots highlight outliers with circles and asterisks.

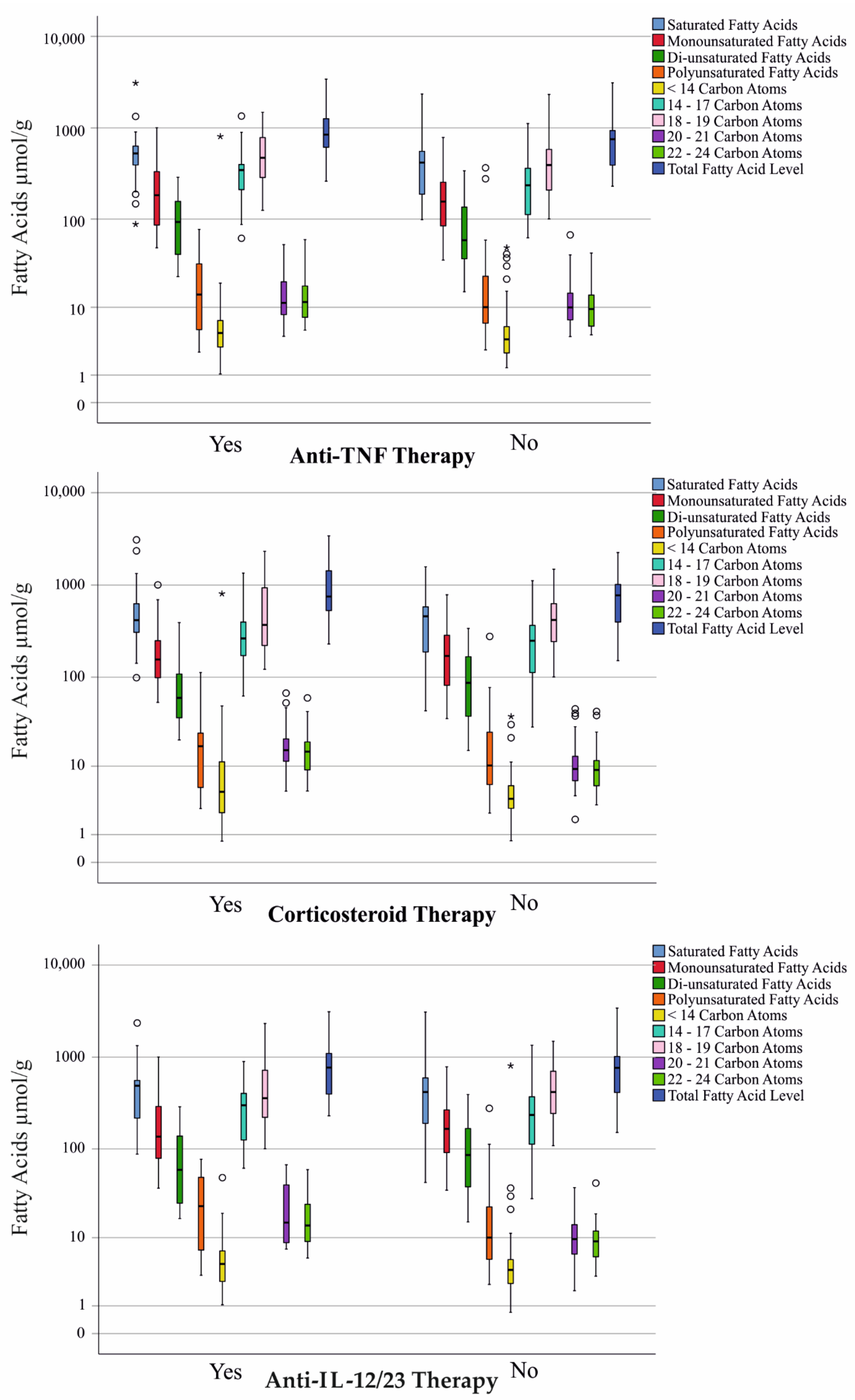

Figure S3. Saturated, monounsaturated, di-unsaturated and polyunsaturated fatty acid levels and fatty acid levels classified according to the number of carbon atoms of patients with different therapies. There were no differences between the groups. Boxplots highlight outliers with circles and asterisks.
